# What Is Authentic Maple Water? A Twelve-Month Shelf-Life Study of the Chemical Composition of Maple Water and Its Biological Activities

**DOI:** 10.3390/foods12020239

**Published:** 2023-01-04

**Authors:** Kara J. Torrey, Yongqiang Liu, Huifang Li, Hang Ma, Christopher W. Via, Matthew J. Bertin, Navindra P. Seeram

**Affiliations:** Department of Biomedical and Pharmaceutical Sciences, College of Pharmacy, University of Rhode Island, Kingston, RI 02881, USA; kara_torrey@uri.edu (K.J.T.); yongqiang_liu@uri.edu (Y.L.); huifang_li@uri.edu (H.L.); hang_ma@uri.edu (H.M.); christopher_via@my.uri.edu (C.W.V.); mbertin@uri.edu (M.J.B.)

**Keywords:** maple water, shelf-life, chemical composition, standards of identity, antioxidant, anti-inflammatory

## Abstract

Maple water (maple sap) products are produced from sap tapped directly from maple trees, but there is confusion and lack of industry consensus and consumer knowledge as to what constitutes ‘authentic’ maple water. With an immense potential for growth in the multi-billion dollar functional beverage market, the market promotion of maple water products hinges on establishing standards of identity (SI), which are currently lacking. Herein, we aim to provide publishable SI and compositional chemistry findings of maple water. The chemical composition (including polyphenols, sugars, amino acids, and organic acids) of a pasteurized maple water was monitored over a 12-month (at 0, 4, 8, and 12 months) shelf-life. Furthermore, LC-MS/MS and molecular networking-based methods were developed to identify the phytochemical profile of a maple water extract (MWX) and to compare it to a previously chemically characterized phenolic-enriched maple syrup extract (MSX). Both MSX and MWX have similar phytochemical profiles and chemical characteristics. In addition, MSX and MWX showed moderate antioxidant capacity (in free radical scavenging and anti-tyrosinase assays) and anti-inflammatory effects (in soluble epoxide hydrolase and cyclooxygenase-2 inhibition assays). Our findings provide critical information on the SI and stability (in chemical composition) of maple water, which will help define, authenticate, and distinguish it from other functional beverages, thereby positioning the maple industry for promotion and growth in this market sector.

## 1. Introduction

Maple water is the clear watery sap (containing 95–99% water) obtained directly from maple (*Acer*) trees. Whilst traditionally maple water (used henceforth to refer to maple sap obtained directly from maple trees) was consumed by the indigenous people of North America as a ‘medicinal tonic’, its primary use has been for the production (by boiling) of maple syrup [1]. However, in recent years, commercial maple water products have been launched in the functional beverage market sector. These new maple water beverage products have attracted the interest of consumers seeking functional foods that may contain natural ingredients associated with health benefits, and by consumers seeking alternatives to soda. The potential market for this new type of water could become as popular as coconut water, reaching millions in sales, without affecting the more traditional maple syrup market. Notably, in the past few years, several human clinical studies have been published investigating the potential health benefits of maple water products [2,3,4,5], supporting increasing research interest in these beverages. The market development and promotion of maple water products is long overdue as maple water was identified as a value-added product with immense growth potential for the maple industry. With health and wellness being an area of growing concern in developed countries, the entire maple market can benefit from increased consumer awareness and diversification of maple products.

Over the past decade, maple syrup has gained popularity as a ‘smarter sweetener’, due to extensive scientific research (www.uri.edu/maple; accessed on 3 November 2022). Our group has initiated a research program to systematically study the phytochemical constituents and biological activities of maple foods including maple syrup, maple sugar, and maple water, as well as their derived extracts. This led to a series of publications reporting maple syrup’s chemical composition, including sugars (mainly sucrose), organic acids, amino acids, minerals, complex carbohydrates, and phytochemicals [1,6,7,8,9,10,11]. To date, over 60 phytochemicals have been identified in maple-derived foods. The majority of the isolated phenolic compounds are of the lignan subclass (>20), which represent characteristic ‘maple chemical standards’ [7,8]. Notably, a product-specific standard, namely MaPLES (maple phenolic lignan-enriched standard), was developed for phenolic quantification of maple-derived foods [12]. In addition, a panel of other phenolic compounds including coumarin, gallic acid derivative, phenylpropanoid, and stilbene subclasses, as well as sesquiterpenes, have been isolated and identified from maple foods [11,13,14,15]. A phenolic process-derived compound, named quebecol, has also been previously identified and isolated by our group [6].

Functional beverages are rapidly growing in popularity, driven by increasing consumer demand for natural and locally produced foods with added health benefits [16]. Maple sap beverages in particular have become rather popular on the U.S. market, due to their complex nutrient matrix [17]. In fact, the current maple water market is estimated to be roughly USD 20 million, and is predicted to reach over USD 2 billion by 2025 according to market research [18]. Our group has previously conducted a study to analyze the phytochemical profile of pasteurized maple sap, showing that the phenolic compounds present in maple sap are preserved after the heating processes involved in pasteurization and sterilization [10]. With chromatographic and spectrophotometric analysis, it was revealed that other chemical components, including sugars [19], amino acids [10,20], organic acids [10,21], and minerals [10,22], were present in various sap samples [17]. However, the stability of these chemical standards of maple water over storage time is unknown. Herein, we conducted this study to evaluate the stability of maple water chemical composition over a 12-month period (at 0, 4, 8, and 12 months) of shelf-life. In addition, liquid chromatography (LC) tandem mass-spectrometry (LC-MS/MS) methods were developed to identify the phytochemical constituents of a maple water extract (MWX) and to compare it to that of a maple syrup extract (MSX), which was previously developed by our group [9]. The biological activities, including the antioxidant and anti-inflammatory effects of MSX and MWX, were evaluated using in vitro enzymatic assays.

## 2. Materials and Methods

### 2.1. Chemicals and Reagents

HPLC and LC-MS grade methanol and water were obtained from Wilkem Scientific (Pawtucket, RI, USA). Ethyl acetate, acetonitrile, formic acid, and sodium carbonate were purchased from Sigma-Aldrich (St. Louis, MO, USA).

### 2.2. Maple Water

Maple water samples were provided by Drink Simple (Saint Albans, VT, USA) by kind courtesy of the co-founder, Kate Weiler. The maple water samples were from the same batch/lot number and were shipped to the University of Rhode Island (Kingston, RI, USA) and immediately stored at a constant temperature (4 °C) for the entire period of the shelf-life study. Three individual boxes of maple water (n = 3) were used for the measurements of physiochemical and chemical characteristics.

### 2.3. Physiochemical Properties of Maple Water

Physiochemical properties including the pH and degree Brix (°Bx) of maple water (three samples for each test) were measured according to methods previously reported [23,24]. Briefly, the pH of maple water was measured using an Orion Star pH Conductivity Meter (ThermoFisher Scientific; Waltham, MA, USA), using 2 mL of sample for each measurement. Prior to the analysis, the pH meter was calibrated with standard buffer solutions of pH 7.00, 4.01, and 10.01. The overall sugar content was measured by refractometry using an Aichose 0–80% °Brix Meter Refractometer, which was calibrated with deionized water, followed by measuring 200 µL of maple water in triplicate. Readings were expressed as °Bx at 20 °C.

### 2.4. Sugar Contents of Maple Water by HPLC-Refractive Index (RI)

Specific sugar contents including sucrose, glucose, and fructose of maple water were determined by an HPLC-RI method following a procedure reported by our group [10]. The HPLC-RI analyses were conducted with a Waters Sugar-Pak I column (300 × 6.5 mm, 10 μm; Waters Corp.; Milford, MA, USA) kept at a column temperature of 90 °C. Samples were analyzed with an isocratic solvent system consisting of ethylenediaminetetraacetic acid calcium disodium salt (EDTA; 50 mg/L) over 15 min and a flow rate of 0.60 mL/min with an injection volume of 20 μL. Each maple sap sample (20 μL) was filtered through a 13 mm diameter sterile syringe filter with a 0.45 µm pore size (Millex-HV Syringe Filter from MilliporeSigma; Burlington, MA, USA) before it was injected for HPLC-RI analyses in triplicate. Sugar peaks of sucrose, glucose, and fructose were identified on the basis of a comparison of their HPLC-RI retention times to the respective standards.

### 2.5. Measurement of the Amino Acid Content of Maple Water by HPLC-Fluorescence (FL)

The levels of the major amino acids in maple water including glutamine (GLU), glycine (GLY), and arginine (ARG) were determined by an HPLC-FL assay using an AccQ Tag and AccQ Fluor Reagent Kit (Waters Corp Milford, MA, USA). The HPLC-FL analyses were performed using a Waters AccQ Tag column (150 × 3.9 mm; column temperature of 40 °C) with a flow rate of 0.70 mL/min and an injection volume of 5 μL. The FL detector used a detection wavelength of Ex of 250 nm and Em of 395 nm. A linear gradient solvent system consisting of solvent A (AccQ Tag Eluent A Concentrate; 10:1 dilution) and solvent B (60% acetonitrile in water) was used as follows: 0 min, 100% A; 0.5 min, 98% A; 15 min, 93% A; 19 min; 90% A; 30 min, 80% A; 40–45 min; 64% A to 100% B; 46 min, 100% A. The amino acid peaks were identified by a comparison to their respective amino acid standards.

### 2.6. Organic Acid Content of Sap Samples by HPLC-DAD

The major organic acids in the maple water, including malic acid and fumaric acid, were determined by HPLC-DAD analyses, as previously reported [10]. An Allure^®^ organic acid column (150 × 4.6 mm; Restek Corp., Bellefonte, PA, USA; column temperature of 25 °C) with a flow rate of 0.50 mL/min and an injection volume of 20 μL was used for the analyses. The isocratic solvent system consisted of 100 mM KH_2_PO_4_ over 30 min. The organic acid peaks were identified on the basis of a comparison of their retention times to the organic acid standards.

### 2.7. Mineral Content of Maple Water by ICP-MS

The predominate mineral contents of the maple water, including Mg and Mn, were measured by an inductively coupled plasma mass spectrometry (ICP-MS) method, as we previously reported [10]. All the analyses were conducted on a Thermo Scientific iCAP Qa instrument coupled to an ASX-510 HS High Speed autosampler (Thermo Scientific, West Palm Beach, FL, USA). The maple water sample (10 μL) was mixed with nitric acid (1 mL; ICP-MS grade) in a reaction vessel followed by digestion in a microwave. Then, the sample (400 μL) was further diluted with DI water for ICP-MS analyses.

### 2.8. Preparation of Maple Extracts

A standardized food grade phenolic-enriched maple syrup extract (MSX) was produced as previously reported [9], and is readily available in our laboratory. In brief, the MSX was previously produced in a pilot-scale facility using adsorption grade chromatography with an FDA food-grade approved resin (Amberlite XAD-16) and food-grade solvents (water and denatured ethanol). MSX presents as a dark brown free-flowing dried powder after solvent removal. A maple water extract (MWX) was developed from pasteurized maple sap samples provided by a local maple water retailer, Drink Simple (Saint Albans, VT, USA). The maple sap was tapped mainly from sugar maple (*Acer saccharum*) trees, along with few red maple trees (*Acer rubrum*) from Vermont and upstate New York regions. After collection, the raw sap was pasteurized to prevent bacterial growth and contamination before packaging. The sap samples were shipped to our laboratory and stored at 4 °C until needed for chemical and biological evaluation. A volume of 4.615 L of maple water was used to prepare an MWX. The pasteurized maple sap was subjected to liquid–liquid partitioning with ethyl acetate (×3 each volume). Ethyl acetate was chosen as the extracting solvent due to its use previous methods with high recovery of phenolic compounds for maple sap and syrup extracts. The combined ethyl acetate extracts were collected and concentrated using a rotatory evaporator. Upon weighing, the resulting ethyl acetate extract yield was 207.3 mg.

### 2.9. Total Polyphenol Content by Folin-Ciocalteau Method

Phenolic contents of plant foods are commonly quantified by the Folin–Ciocalteau (F–C) assay, based on gallic acid equivalents (GAEs) [12]. Briefly, the samples (MSX and MWX) were dissolved in 2 mL of aqueous methanol (50%). An amount of 200 µL of each sample was incubated with 3 mL of the aqueous methanol and 200 µL of the F–C reagent for 10 min at 25 °C. Then, 600 µL of 20% sodium bicarbonate (Na_2_CO_3_) solution was added to each tube and mixed vigorously. Tubes were then incubated for an additional 20 min in a 40 °C water bath and then immediately cooled in an ice bath to room temperature. Samples and standards (gallic acid) were processed identically. The UV absorbances were measured on a SpectraMax plate reader (Molecular Devices, San Jose, CA, USA) at a wavelength of 755 nm.

### 2.10. LC-MS/MS Analysis and Molecular Networking

Liquid chromatography coupled with mass spectrometry (LC-MS) was used to compare the chemical profile of MWX to MSX. Tandem mass spectrometry (LC-MS/MS) was performed using a ThermoFisher Scientific LTQ XL linear ion trap mass spectrometer with an electrospray ionization (ESI) source. Analytical HPLC was carried out using a Dionex UltiMate 3000 HPLC system equipped with a micro vacuum degasser, an autosampler, and a diode-array detector [25]. Samples were separated on a Phenomenex Luna C_18_ column (5 µm; 150 × 4.6 mm). The mobile phase (A) consisted of (*v*/*v*) formic acid in water % and (B) 0.1% (*v*/*v*) formic acid in acetonitrile with a gradient condition as follows: 5% A from 0 to 5 min, 5–16% A from 5 to 16 min, and 16–26% A from 16 to 76 min. The column temperature was 40 °C with a flow rate of 0.5 mL/min and the injection volume was 20 µL. The MS analyses were performed in positive and negative modes, separately. The MS spray voltage was 3.50 kV for the negative mode and 5.0 kV for the positive mode, with a capillary temperature of 275 °C. Following the MS/MS fragmentation, raw data files were converted to a mz XML format using MSConvert from the ProteoWizard suite (http://proteoqizard.sourceforge.net/tools.shtml; accessed on 15 October 2021). The molecular networking was generated using the online platform at the global natural products social molecular networking (GNPS) website (gnps.ucsd.edu). The data were filtered by removing all MS/MS fragment ions within 17 Da of the precursor *m*/*z*. The MS/MS spectra were window filtered by selecting the top six fragment ions in the 50 Da window throughout the spectrum. The precursor ion mass tolerance was set to 2.0 Da and the MS/MS fragment ion tolerance was set to 0.5 Da. A network was then created where edges were filtered to have a cosine score above 0.4 and more than four matched peaks. Next, edges between two nodes were kept in the network if each of the nodes appeared in each other’s respective top 10 most similar nodes. Finally, the maximum size of the molecular family was set to 100 and the lowest scoring edges were removed from molecular families until the molecular family size was below this threshold. The spectra in the network were then searched against the GNPS spectral libraries. The library spectra were filtered in the same manner as the input data. All matches kept between network spectra and library spectra were required to have a score above 0.5 and at least four matched peaks [26]. The network was visualized using the Browser Network Visualizer tool available on the GNPS website and then imported into the program Cytoscape for additional analysis.

### 2.11. Antioxidant Activity by Free Radicals (DPPH) Scavenging Assay 

The antioxidant potential of MWX was determined using a DPPH assay [27]. Test samples including MSX, MWX, and a representative compound from MSX, namely, quebecol, were assayed at various concentrations in a 96-well plate in triplicate, as we previously reported.

### 2.12. Tyrosinase Inhibition

The anti-tyrosinase potential of MSX and MWX were determined using a reported method [28,29]. Briefly, the assay was performed in a 96-well microplate in triplicate. Samples were dissolved in methanol and diluted to different concentrations with phosphate buffer (0.1 M; pH 6.8). Each well contained a total volume of 200 µL reactants, consisting of 40 µL of tyrosinase enzyme (100 units/mL), 40 µL L-tyrosine (2.5 mM), 80 µL of assay buffer, and 40 µL of test the sample. The plate was incubated at room temperature for 30 min and then the absorbance of each well was measured at a wavelength of 490 nm. The percentage of tyrosinase inhibition was calculated as follows: [(A_control_ − A_sample_)/A_control_] × 100%.

### 2.13. Anti-Inflammatory Assays (Soluble Epoxide Hydrolase and Cyclooxygenase-2 Inhibition Assays)

The anti-inflammatory effects of MSX and MWX were determined by two enzymatic models including the soluble epoxide hydrolase (sEH) and cyclooxygenase-2 (COX-2) inhibition assays. A sEH inhibitor screening assay kit (Cayman Chemical; Ann Abor, MI, USA) was used to evaluate the enzyme inhibitory potential of the selected compounds in triplicate. This kit employs human recombinant sEH and (3-phenyl-oxiranyl)-acetic acid cyano-(6-methoxy-naphthalen-2-yl)-methl ester (PHOME) as the substrate. The enzyme inhibition was measured by a fluorescent readout at an excitation and emission wavelength of 330 nm and 465 nm, respectively. MSX, MWX, and quebecol were tested in triplicate and 1-aryl-3-(1-acylpiperidin-4-yl)urea (a potent sEH inhibitor) was used as the positive control. The COX-2 inhibitory effect of MSX and MWX was assessed with a cyclooxygenase fluorescent inhibitor screening assay kit (Cayman Chemical, Ann Arbor, MI, USA). Briefly, the reaction between prostaglandin G2 (PGG2) and 10-acetyl-3,7-dihydrophenoxazine (ADHP), which produces a highly fluorescent compound resorufin, was analyzed at an excitation wavelength of 530 nm and emission wavelength of 585 nm using a SpectraMax Plate Reader (Molecules Devices, San Jose, CA, USA). DuP-697 was used as a positive control for COX-2 inhibition assay. The plate was incubated for 5 min at room temperature before adding 10 µL of ADHP to the test samples followed by initiating the reaction with the addition of 10 µL of arachidonic acid. The plate was incubated for 2 min at room temperature and then the plate was read at an excitation and emission wavelength of 535 nm and 590 nm, respectively.

### 2.14. Statistical Analysis

Data from the measurements of the characteristics of maple water samples are presented as mean ± standard deviation (S.D.) of triplicated experiments. Statistical analysis was performed with GraphPad Prism9 (GraphPad Software, La Jolla, CA) using a one-way analysis with repeated measures, ANOVA, and a variance with multiple comparisons to the starting point. Significance for all tests was noted if *p* ≤ 0.05 (*), *p* ≤ 0.01 (**), *p* ≤ 0.001 (***), and *p* ≤ 0.0001 (****).

## 3. Results

### 3.1. Maple Water’s Total Phenolic Content, °Bx, and pH Maintained for 12 Months

**Figure 1:**
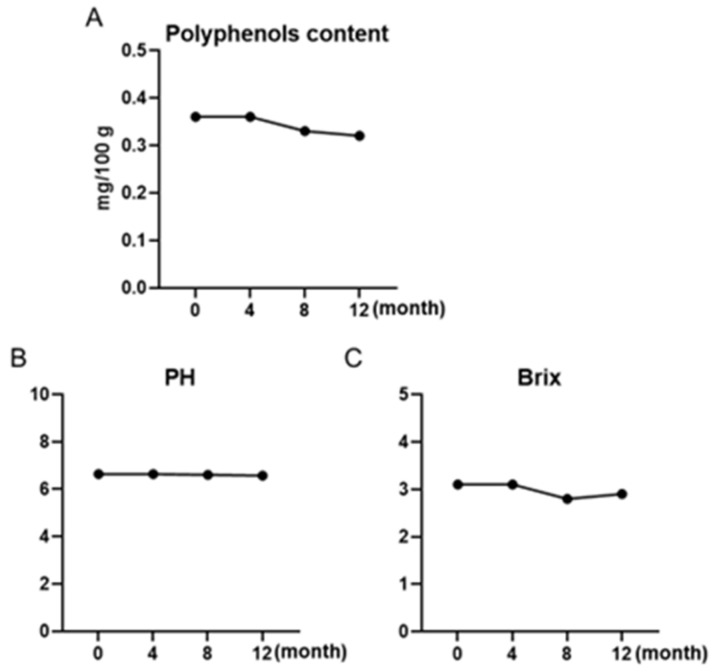
Changes in maple water total phenolic content (**A**), °Bx (**B**), and pH (**C**) during a storage time of 12 months at room temperature. Each sample was measured at time points of 0, 4, 8, and 12 months.

The maple water samples at a series of shelf-life time points (0, 4, 8, and 12 months) were assessed for changes in phenolic content, pH, and °Bx. The level of total phenolics of maple water remained identical over the tested shelf-life period (0.36, 0.36, 0.33, and 0.32 mg/100 g of the GAE at 0, 4, 8, and 12 months, respectively), which was similar to pasteurized and sterilized maple sap (GAE = 0.27 mg/100 g), as we previously reported [10]. The pH values of maple water samples at all time points were maintained in the range between 6.57 and 6.63 (Figure 1), which was in agreement with the reported pH value (up to 6.65) of maple sap from eastern Canada [30]. The pHs of maple water at 0 and 4 months were 6.3 and 6.64, and then slightly decreased to 6.61 and 6.57 at 8 and 12 months, respectively. Similarly, the °Bx values of maple water samples at the first two timepoints were 3.1, which then slightly decreased to 2.8 and 2.9 at 8 and 12 months, respectively. The Brix value of maple water was slightly higher than the reported values of a set of maple sap samples (1.81–2.21) collected from different seasons [21].

### 3.2. Sugar Contents of Maple Water Slightly Decreased over a 12-Month Shelf-Life

**Figure 2:**
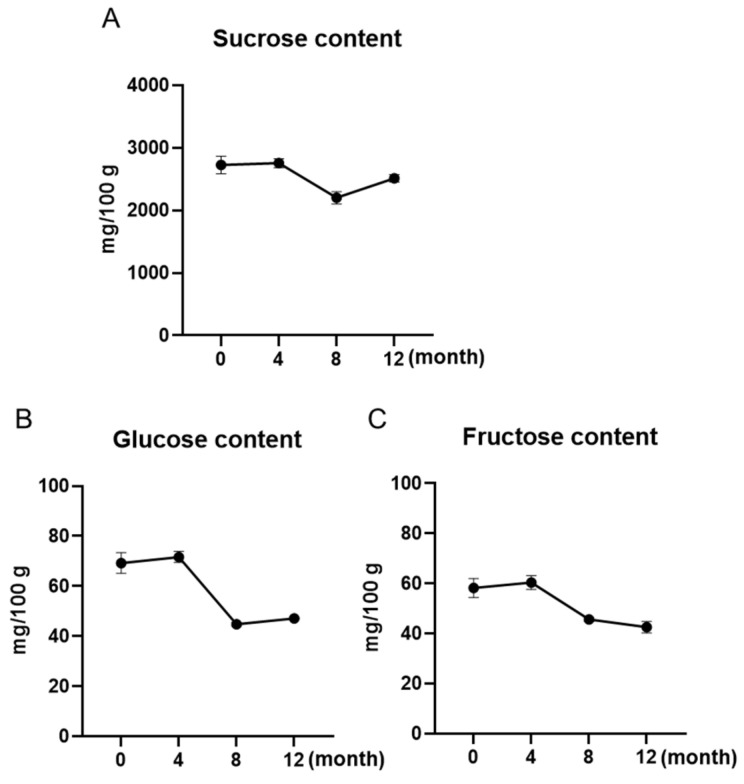
Changes in maple water carbohydrate content including sucrose (**A**), glucose (**B**), and fructose (**C**) during a storage time of 12 months at room temperature. Each sample was measured at time points of 0, 4, 8, and 12 months.

We further analyzed the changes in maple water in individual sugar contents (sucrose, glucose, and fructose) at different time points. As shown in Figure 2A, sucrose was the predominant type of sugar in maple water and its levels were 2752.0 and 2753.3 mg/100 g at 0 and 4 months, respectively. The levels of sucrose slightly decreased to 2200.3 and 2512.0 mg/100 g at further time points (8 and 12 months). This pattern was similar to the levels of the other sugars, glucose and fructose, which had higher levels at the first two time points (69.2 and 71.7 mg/100 g; and 58.2 and 60.4 mg/100 g) and then lowered to 44.8 and 47.1 mg/100 g and 45.6 and 42.5 mg/100 g at the latter time points, respectively (Figure 2B,C). This observation was in agreement with a reported study showing that sucrose is the major sugar in maple water (ca. 2100 mg/100 g) [21].

### 3.3. Other Chemical Contents of Maple Water Maintained over a 12-Month Shelf-Life

**Figure 3:**
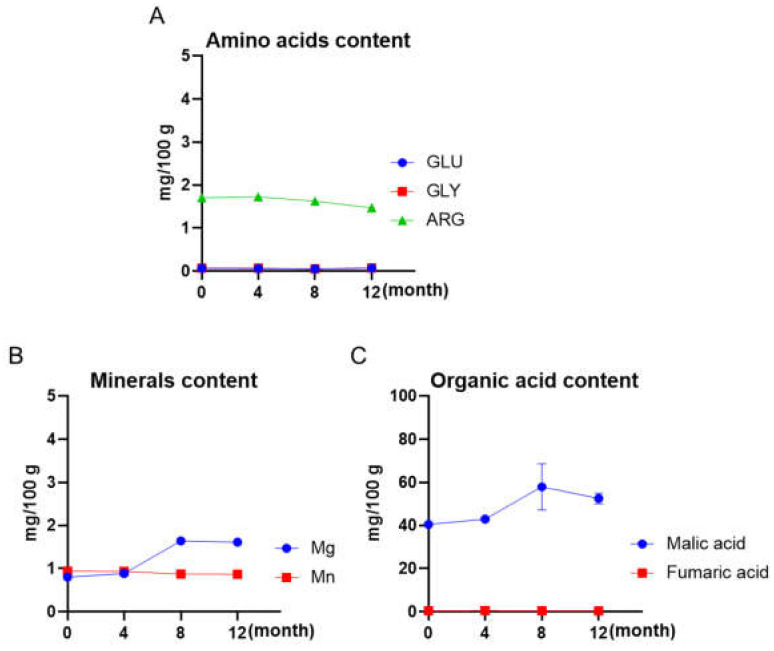
Changes in maple water chemicals including amino acids (GLU, GLY, and ARG) (**A**), minerals (Mg and Mn) (**B**), and organic acids (malic acid and fumaric acid) (**C**) during a storage time of 12 months at room temperature. Each sample was measured at time points of 0, 4, 8, and 12 months.

The amino acid contents of maple water remained stable over a 12-month shelf-life time. The levels of the most abundant amino acid (i.e., Arg) were 1.7, 1.7, 1.6, and 1.6 mg/100 g at the time points of 0, 4, 8, and 12 months, respectively (Figure 3A). The other two amino acids, Glu and Gly, were detected at much lower levels (0.07 and 0.07 mg/100 g at the first time points, respectively) and remained at an identical level for the entire 12-month period. This observation is in agreement with our previously reported data on the level of amino acids in pasteurized and sterilized maple water [10]. Next, the levels of minerals (Mg and Mn) and organic acids (malic acid and fumaric acid) in maple water were examined at different time points. The mineral contents remained stable, as the Mg levels ranged from 0.8 to 1.6 mg/100 g (0 to 12 months), respectively, whilst the Mn levels were identical for all time points (0.9 mg/100 g at 0, 4, 8, and 12 months; Figure 3B). The levels of an organic acid in maple water, namely malic acid, were 40.5, 42.9, 57.8, and 52.5 mg/100 g at 0, 4, 8, and 12 months, respectively, whereas the levels of fumaric acid were negligible during the entire 12-month shelf-life (Figure 3C). The levels of chemical contents including amino acids, minerals, and organic acids were comparable to values in studies from our group [10] and others [21,22].

### 3.4. Maple Food Extracts (MSX and MWX) Had Comparable Phenolic Content

To further study the chemical composition and biological effects of maple food extracts, MWX was prepared and compared with MSX. Polyphenols, widely studied for their antioxidant properties, are known to be the major phytochemicals in maple syrup and maple food products. MSX showed an average of 40.35% phenolic content, whereas MWX showed an average of 32.01% (Table 1), which was in agreement with our previously reported values [9,10].

### 3.5. LC-MS/MS Analysis of Maple Food Extracts

The implementation of well-established analytical methods, such as LC-MS/MS, is essential for the chemical analysis of botanical food extracts. Given limited phytochemical MS compositional data available for pasteurized maple sap samples, which are necessary for its future advancement as a functional beverage, we employed LC-MS/MS methods to identify the compounds present in MWX with a comparison of our group’s previously characterized phenolic-enriched maple syrup extract (MSX) [9]. A LC-M/MS analysis of the compounds present in the maple food extracts (MWX and MSX) was performed in an ESI positive ionization mode, and all of the compounds showed [M + H]^+^ or [M + Na]^+^ ions (Table 2). Low-resolution mass data were then acquired in full scan analysis and product ion mass data were acquired by the information-dependent acquisition method. A total of eight compounds were successfully characterized and/or dereplicated in the maple food extracts: seven in both MSX and MWX, whilst one was only found in MSX. Accurate mass measurements, errors (in ppm), molecular class, and the main MS/MS product ions for all the identified compounds are summarized in Table 2.

**Figure 4:**
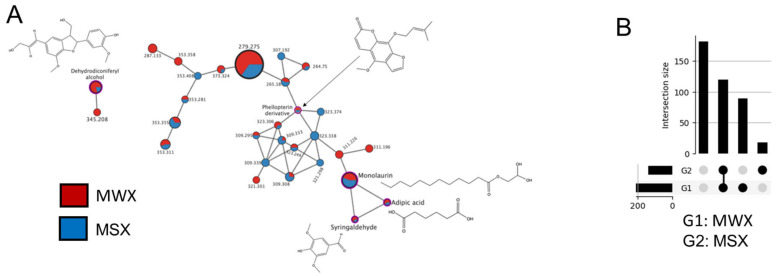
(**A**) An LC-MS/MS-based molecular network of chemicals from MSX and MWX using the GNPS platform and Cytoscape. Red nodes represent compounds present in MWX, while blue nodes represent compounds present in MSX. Compounds present in both extracts (red and blue) are shown in pie percentages out of a total quantification (100%) in both extracts. The size of each node is relative to the quantification amount of each compound based on ion count. Five total compounds were able to be dereplicated through molecular networking: dehydrodiconiferyl alcohol, a phellopterin analogue, monolaurin, adipic acid (adipate), and syringaldehyde. (**B**) UpSet plot detailing unique metabolite features found in MWX (G1) and MSX (G2). Intersection size shows the features attributable to each sample or found in both samples.

To further identify and visualize the chemical constituents of MSX and MWX, we employed a molecular networking tool with the GNPS platform, an open access tool for specialized metabolite organization and sharing of raw, processed, or identified MS/MS data. The resulting GNPS analysis from MSX and MWX MS data showed 258 library hits. Several of those library hits were further elucidated after filtering through the results matching the *m*/*z* values and MS2 fragmentation patterns. Five of the six dereplicated compounds from GNPS that were present in both MSX and MWX were (1) 2-amino-1,9-dimethyl-6,9-dihydro-1H-purin-6-one, (2) monolaurin, (3) adipic acid, (4) decanedioic acid, bis(2-ethylhexyl) ester, and (5) a phellopterin analog. The final sixth compound, (-) catechin, was only found to be present in MSX (Table 2). To obtain a visual representation of the results of all library hits, a molecular network was created. Molecular networking is a visual tool used to represent the chemical space in given MS experiments by showing correlation in fragmentation patterns from related molecules. The GNPS analysis revealed 10 different molecular networks, with the largest network containing a total of 27 nodes (Figure 4). The resulting network showed a panel of analogues present in MSX or MWX separately, as well as analytes present in both. Red nodes represent compounds present in MSX, while blue nodes represent compounds present in MWX. Compounds present in both extracts (red and blue) are shown in pie percentages out of a total quantification (100%) in both extracts. The relative size of each node is representative of the amount of the detected compound from ion count data. Five compounds found in the network were successfully dereplicated. A small cluster in the lower right corner shows the similarity in structure and fragmentation patterns of monolaurin, adipic acid, and syringaldehyde (Figure 4). Monolaurin and adipic acid were library hits from the GNPS platform, where their *m*/*z* values were about 1 ppm different than their expected values (Table 2). Syringaldehyde was identified by its *m*/*z* value of 182.943 [M + H]^+^ and its presence was found in both MSX and maple water [9,10]. A phellopterin analog was identified with an *m*/*z* value of 337.34 [M + Na]^+^, likely with an additional methyl (CH_3_) added to one of the aromatic oxygens. Dehydrodiconiferyl alcohol, a lignan, was the fifth molecule identified, with an *m*/*z* value of 359.181 [M + H]^+^. This lignan is also known to be present in both MSX and maple water [9,10]. The other remaining 22 nodes in the network represent compounds present in the maple food extracts that were unable to be dereplicated, where they are labeled by their precursor *m*/*z* values.

### 3.6. Maple Food Extracts Showed Promising Antioxidant Activity

**Figure 5:**
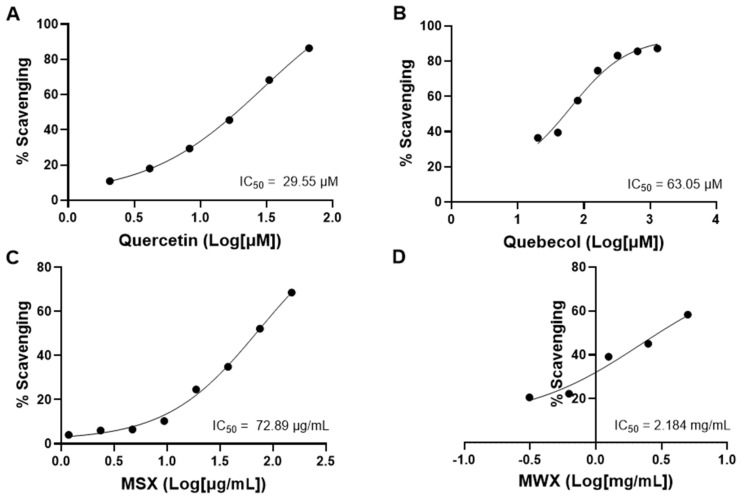
Antioxidant effects of a phenolic free radical scavenger quercetin (**A**), a unique phenolic named quebecol isolated from maple syrup (**B**), MSX (**C**), and MWX (**D**) evaluated by the DPPH assay.

Maple food extracts including MSX and MWX were evaluated for their antioxidant capacity by DPPH assay. At an initial screening (concentration of 1 mg/mL), MSX and MWX scavenged 74.06 and 20.22% of the DPPH free radicals, respectively. Next, a series of concentrations were used to assess the IC_50_ values of MSX’s and MWX’s free radical scavenging effects. In addition, two positive controls, namely quercetin (a common natural antioxidant) and quebecol (a unique phenolic compound isolated from MSX [6]), were evaluated by DPPH assay. Quercetin and quebecol showed an IC_50_ value of 29.55 and 63.05 μM, respectively (Figure 5A,B). MSX and MWX had IC_50_ values of 72.89 μg/mL and 2.18 mg/mL, respectively (Figure 5C,D).

**Figure 6:**
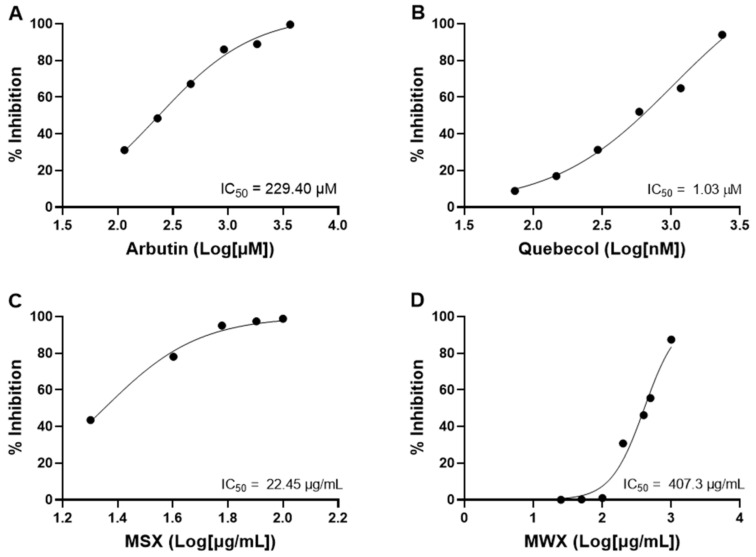
Antioxidant effects of arbutin (**A**), quebecol (**B**), MSX (**C**), and MWX (**D**), evaluated by a tyrosinase inhibition assay.

Furthermore, the antioxidant activity of maple food extracts was evaluated by a tyrosinase inhibition assay, which measures the inhibitory effect on the oxidation of the diphenol moiety to the corresponding O-quinone. An initial anti-tyrosinase activity screening of the maple food extracts revealed that MSX and MWX inhibited tyrosinase activity by 76.07 and 81.74%, respectively. Further evaluations of MSX and MWX, along with positive controls arbutin (a known tyrosinase inhibitor) and quebecol, were performed at a series of concentrations to obtain the IC_50_ values of inhibition. Among the maple food extracts, MSX showed a lower IC_50_ value (22.45 µg/mL) compared to MWX (IC_50_ = 407.30 µg/mL), whereas arbutin and quebecol showed IC_50_ values of 229.4 and 1.03 µM, respectively (Figure 6). Based on data from two antioxidant assays, MSX showed superior antioxidant potential to MWX.

### 3.7. Maple Food Extracts Exerted Moderate Anti-Inflammatory Activity

The anti-inflammatory activity of MSX and MWX was evaluated in enzymatic models including soluble epoxide hydrolase (sEH) and cyclooxygenase-2 (COX-2) inhibition assays. These two assays were first validated by testing the positive controls: quercetin (at 125 µM) had an inhibition rate of 74.17% on sEH and celecoxib inhibited COX-2 by 66.5% at 0.45 µM. Then, MSX and MWX were evaluated in these assays at a concentration of 100 µg/mL. MSX showed a potent anti-sEH activity with an inhibition of 91.53% and a weak inhibition on COX-2 (10.97%). MWX only showed weak inhibitory effects on sEH and COX-2 with an inhibition of enzyme activity of 6.27% and 21.87%, respectively (Table 3).

## 4. Discussion

Although maple water products are predicted to gain a multi-billion dollar revenue on the functional beverage market, their market promotion and sales have been hampered by the lack of SI for this natural product. Efforts are needed to educate the consumer about what maple water is composed of, which is not syrup, but a water-based beverage with nutrients and hydration benefits. Based on current scientific knowledge, the chemical composition of different brands of maple water products may vary depending on the source of the raw sap and their packaging treatments [17]. Indeed, maple water is a natural product susceptible to alteration, adulteration, and falsification. For instance, adulteration can be accomplished by adding foreign sugars to maple water products or by diluting maple syrup with water which may constitute fraud if labelled and sold as pure and natural maple water. Moreover, there is confusion about whether “permeate” (reverse osmosis water obtained during maple sap boiling which is devoid of nutritional value) is authentic maple water. This confusion is further compounded since consumers commonly think of ‘sap’ as being ‘sticky’ and ‘syrupy’ and not a water-based product. Therefore, because of consumer confusion and lack of validated industry standards, the establishment of SI for maple water products, supported by validated chemical composition analyses, and the communication of this knowledge to consumers, is critical to avoid this great market opportunity being missed.

The constituents of natural maple water include macronutrients, micronutrients, and phytochemicals (‘phytonutrients’), namely sucrose, complex polysaccharides, vitamins, minerals, amino acids, organic acids, phytohormones, and polyphenolic antioxidant compounds [6,7,10,14,15]. However, due to its largely ‘watery (95–99%) nature’, it is not surprising that once maple water is collected, it serves as a rich nutrient medium for the growth of microorganisms [1]. Therefore, maple water products should be pasteurized or sterilized to eliminate microbial growth to ensure a desirable shelf-life that is appropriate for human consumption. However, whether this practice affects the shelf-life of maple water in terms of its chemical composition is unknown. To date, our group has conducted the only published study to evaluate the chemical composition of maple water after pasteurization and sterilization, wherein we reported that the variety of substances found naturally in sap are largely preserved in these processes [10]. Notably, maple water collected at different periods of the ‘tapping’ season over different years from different locations has been reported to show considerable variability in sucrose, organic acid (malic, acetic, and lactic acids), mineral (K, Ca, and Mg), and phenolic compound contents [21]. In the current study, we examined whether a 12-month storage period impacts the chemical composition of a selected maple water product. Based on the analyses of the maple water product’s chemical characteristics, including °Bx, pH, total polyphenols, sugar content, mineral level, organic acids, and amino acids content, maple water in a Tetra Pak^®^ container remained stable over 12 months (i.e., changes in these chemical levels were not statistically significant). This is similar to the results of a published comparative study that was conducted on coconut water, which revealed that the pH values of coconut water samples varied and increased slightly [31].

Maple foods share a unique phytochemical profile, in which the lignan-type of phenolics is predominant. This is a characteristic SI for the authentication of maple water. Given the limited phytochemical MS compositional data available on maple water samples, which are critical for its market advancement as a functional beverage, we employed LC-MS/MS methods to identify the compounds present in MWX, a pasteurized maple water extract. Although a few phytochemicals, including lignans, were identified in MSX and MWX by the LC-MS/MS method and the network-based chemical analysis, some limitations should be addressed. The inability to dereplicate compounds comes from the limitations of low-resolution MS/MS experiments. In the future, using high-resolution mass spectrometry would dramatically improve the results of the networking results, as there would be an enhanced ionization of each analog in the extracts. Using high-resolution electrospray ionization mass spectrometry (HRESIMS) files would increase the accuracy of the library hits from the GNPS platform especially. These samples were also analyzed in positive mode, where sodium adducts can become an obstacle for dereplication. Moving forward, future work involving liquid-chromatography-tandem mass spectrometry (LC-MS/MS) with the multiple reaction monitoring mode could serve as a next step for a more accurate simultaneous multi-analyte quantitation across these extracts [32]. Employing these types of workflows could provide more accurate mass measurements and high-quality information on targeted phytochemical profiles in these complex maple food extracts. MRM requires the mass spectrometer to only monitor specific *m*/*z* values of interest; therefore, it would allow for the detection of smaller fragments and metabolites of interest. This would have to be performed on a higher resolution instrument such as a triple quadruple or linear ion trap MS [33]. Furthermore, evaluating different extraction methods for maple sap, with the aim of authenticating more metabolites, should be investigated.

Apart from monitoring the shelf-life of maple water, we evaluated the biological effects of maple foods including MSX and MWX, given that phenolic-enriched maple extracts have been reported to exert various biological activities by our research group [34,35,36,37,38,39] as well as by other research groups [40,41,42]. Data from the in vitro antioxidant assays (the DPPH and tyrosinase models) showed that both MSX and MWX are promising antioxidants with free radical scavenging and anti-tyrosinase activities. MSX was more potent than MWX in both antioxidant assays, which suggested that they were not biological equivalent despite their similar phytochemical profiles. This might be due to the presence of different bioactive compounds in these maple food extracts. For example, quebecol, which showed potent DPPH scavenging and anti-tyrosinase effects in the antioxidant assays (Figure 5 and Figure 6), is a unique phenolic compound that has been identified in maple syrup but is not present in maple water [6]. It is possible that compounds generated from the processing of maple water (by intensive boiling) to afford maple syrup conferred different biological activities. A similar trend was observed in the anti-inflammatory models, where MSX was more potent in the sEH inhibition assay but less active in COX-2 inhibition. To date, this is the first study reporting the antioxidant and anti-inflammatory effects of MWX. Further studies are warranted to identify unique compounds in these extracts and evaluate their biological activities. Nevertheless, findings from the biological assays in the current in vitro study showed that phytochemicals in maple food products exerted antioxidant and inflammatory effects, which may partially contribute to the overall beneficial health effects reported in published human clinical studies [2,3,4,5].

## 5. Conclusions

In summary, the phytochemical constituents of MSX and MWX are indeed very similar, as supported by data from our LC-MS/MS analyses and total phenolic content assays. Molecular networking was used as a tool to describe the chemical space in both MSX and MWX. There are likely many more phytochemical compounds contained in both extracts, which provide a chemical basis for SI of maple foods such as maple water. The shelf-life study suggested that maple water’s phytochemical constituents and macronutrients can be stable for a year. The current study allowed us to establish compositional SI that would set authenticity parameters for maple water products to help promote their product integrity, production, and marketability. The molecular networking analysis with LC-MS/MS data facilitated the identification of the unique compositional chemistry of maple water and this is related to its potential antioxidant benefits which will attract and influence consumer attention.

## Figures and Tables

**Table 1:** Total phenolic content of maple food extracts, including MSX and MWX, measured by the Folin–Ciocalteau method.

Sample	Total Phenolic Content (%)			Average (%)
MSX	40.31	40.30	40.43	40.35
MWX	32.38	31.89	31.76	32.01

**Table 2:** Compounds present in maple food extracts, successfully dereplicated, through LC-MS/MS networking using the Global Natural Products Social Molecular Networking (GNPS) platform and previous research. Eight total compounds were able to be dereplicated. The cosine score and shared peaks columns represent the similarity in spectra from the maple food extract(s) and library ‘hit’ spectra from the GNPS platform. * in molecular network.

Compound	Presence	Exact Mass	Spectrum *m*/*z*	Error (ppm)	Adduct	Cosine Score	Shared Peaks	Molecule Class	Identification Source
1,2-amino-1,9-dimethyl-6,9-dihydro-1H-purin-6-one	MSX, MWX	179.08	202.08	<1	[M+Na]^+^	0.91	5	Imidazopyrimidines	GNPS
Monolaurin *	MSX, MWX	274.21	240.21	1.004	[M+H]^+^-H_2_O	0.86	8	Glycerolipids	GNPS
Adipic acid *	MSX, MWX	146.06	165.02	<1	[M+Na]^+^	0.83	5	Fatty acyls	GNPS
Decanedioic acid, bis(2-ethylhexyl) ester	MSX, MWX	426.67	427.43	<1	[M+H]^+^	0.65	4	Fatty acyls	GNPS
Syringaldehyde *	MSX, MWX	182.17	182.94	<1	[M+H]^+^	-	-	Hydroxybenzaldehyde	[9,10]
Phellopterin derivative *	MSX, MWX	300.10	337.34	14	[M+Na]^+^	0.64	6	Coumarins and derivatives	GNPS
Dehydrodiconiferyl alcohol *	MSX, MWX	358.40	359.18	<1	[M+H]^+^	-	-	Lignans	[9,10]
(-) Catechin	MSX	290.08	313.01	<1	[M+Na]^+^	0.62	4	Flavonoids	GNPS; [14,15]

**Table 3:** Anti-inflammatory effects of maple food extracts MSX and MWX in the inhibition assays against soluble epoxide hydrolase (sEH) and cyclooxygenase-2 (COX-2). All samples were tested in triplicate and data are presented as the mean ± SD.

Sample	% Inhibition (100 µg/mL)	
	sEH	COX-2
MSX	91.53 ± 7.4%	10.97 ± 2.5%
MWX	6.27 ± 7.1%	21.87 ± 9.1%

## Data Availability

Raw data obtained in this study are available from the corresponding author upon reasonable request.
